# *MiR-24* Tumor Suppressor Activity Is Regulated Independent of p53 and through a Target Site Polymorphism

**DOI:** 10.1371/journal.pone.0008445

**Published:** 2009-12-24

**Authors:** Prasun J. Mishra, Bo Song, Pravin J. Mishra, Yuan Wang, Rita Humeniuk, Debabrata Banerjee, Glenn Merlino, Jingfang Ju, Joseph R. Bertino

**Affiliations:** 1 Laboratory of Cancer Biology and Genetics, National Cancer Institute, National Institutes of Health, Bethesda, Maryland, United States of America; 2 Department of Pharmacology and Medicine, The Cancer Institute of New Jersey, Robert Wood Johnson Medical School, University of Medicine and Dentistry of New Jersey, Piscataway, New Jersey, United States of America; 3 Translational Research Laboratory, Department of Pathology, Stony Brook University Medical Center, Stony Brook, New York, United States of America; 4 Laboratory of Cellular Oncology, Center for Cancer Research, National Cancer Institute, National Institutes of Health, Bethesda, Maryland, United States of America; Roswell Park Cancer Institute, United States of America

## Abstract

MicroRNAs (miRNAs) are predicted to regulate approximately 30% of all human genes; however, only a few miRNAs have been assigned their targets and specific functions. Here we demonstrate that *miR-24*, a ubiquitously expressed miRNA, has an anti-proliferative effect independent of p53 function. Cell lines with differential p53 status were used as a model to study the effects of *miR-24* on cell proliferation, cell cycle control, gene regulation and cellular transformation. Overexpression of *miR-24* in six different cell lines, independent of p53 function, inhibited cell proliferation and resulted in G2/S cell cycle arrest. *MiR-24* over expression in cells with wt-p53 upregulated TP53 and p21 protein; however, in p53-null cells *miR-24* still induced cell cycle arrest without the involvement of p21. We show that *miR-24* regulates p53-independent cellular proliferation by regulating an S-phase enzyme, dihydrofolate reductase (DHFR) a target of the chemotherapeutic drug methotrexate (MTX). Of interest, we found that a *miR-24* target site polymorphism in DHFR 3′ UTR that results in loss of *miR-24*-function and high DHFR levels in the cell imparts a growth advantage to immortalized cells and induces neoplastic transformation. Of clinical significance, we found that *miR-24* is deregulated in human colorectal cancer tumors and a subset of tumors has reduced levels of *miR-24*. A novel function for *miR-24* as a p53-independent cell cycle inhibitory miRNA is proposed.

## Introduction

MicroRNAs (miRNAs) are small non-coding RNAs, processed from longer transcripts by Drosha and Dicer, that mostly bind to the 3′ untranslated regions (3′UTR) of target genes and inhibit gene expression translationally and/or by destabilizing the target mRNA [1]–[5]. As miRNA expression is altered in many human diseases, including cancer, the discovery of miRNAs has added an entirely new dimension to antitumor therapeutic approaches [6]. Although a few miRNAs are overexpressed in cancer and seem to function as oncogenes themselves (miR-17-92, miR-155), a greater number of miRNAs have been shown to be down-regulated in cancer and have the potential to act as tumor suppressors (i.e., Let-7, miR-15/miR-16, miR-1/miR-206, miR-29, miR-124, miR-143/miR-145) [7]–[9]. Hence miRNAs are differentially expressed in many cancers and play a critical role in oncogenesis [7].

Although miRNAs have been predicted to regulate approximately 30% of all human genes, few miRNAs have been assigned to their target mRNAs and specific functions [10]. *MiR-24* is an abundant miRNA and is well conserved between various species (Fig. S1). *MiR-24* is expressed in normal tissues such as adipose tissue, mammary gland, kidney and in differentiated skeletal muscles [11]. *MiR-24* is found to be upregulated in differentiated cells. High levels of *miR-24* have been reported during post-mitotic differentiation of hematopoietic cell lines [12], during thymic development to naive CD8T cells [13] and during myoblast and neuronal differentiation [14], [15]. *MiR-24* was also found to be upregulated during the stationary phase of growth in CHO-K1 cells [16], and in sodium butyrate differentiated embryonic stem cells [17]. *MiR-24* is also deregulated in Hodgkin lymphoma cell lines [18], and inhibition of *miR-24* in Hela cells markedly increased cell growth [19]. *MiR-24* also plays a role in erythropoiesis by regulating ALK4 and in replicative senescence by regulating p16 [20], [21].

*MiR-24* clusters with two other miRNAs, miR-23 and miR-27, on chromosome 9 (cluster-1: *miR-23b*, *miR-27b and miR-24-1*) and on chromosome 19 (cluster-1: *miR-23a*, *miR-27a* and *miR-24-2*). Deregulations at both sites were found to be associated with CLL [9]. Since *miR-24* is associated with differentiation, we explored the role of *miR-24* in cellular transformation. We have previously shown that *miR-24* has a target site in the 3′UTR of DHFR mRNA and a *miR-24*-*SNP* results in loss of *miR-24*-mediated inhibition of DHFR, in MTX resistance, and is associated with an increase in DHFR mRNA and protein [22]. A recent report also showed that *miR-24* suppressed the expression of cell cycle control genes E2F2 and Myc via binding to 3′-UTR miRNA recognition elements [23].

In this study we used mutant and wild type p53 cell lines to study the effects of *miR-24* on cell proliferation and cell cycle control and their mechanisms of regulation. We demonstrate that *miR-24* regulates cellular proliferation, independent of p53 function, by regulating DHFR expression. Of interest, we found that a *miR-24* target site (TS) SNP 829C→T (hereafter referred as *miR-24-TS-SNP*) in the DHFR 3′ UTR results in loss of *miR-24*-function and high DHFR levels in the cell, and imparts a growth advantage to immortalized cells and induces neoplastic transformation. Of possible clinical significance, we find that *miR-24* is deregulated in human colorectal cancers, and subsets of tumors have reduced levels of *miR-24*.

## Results

### *MiR-24* overexpression, independent of p53-function, inhibits anchorage dependent cellular proliferation and induces G2/S arrest

**Figure 1:**
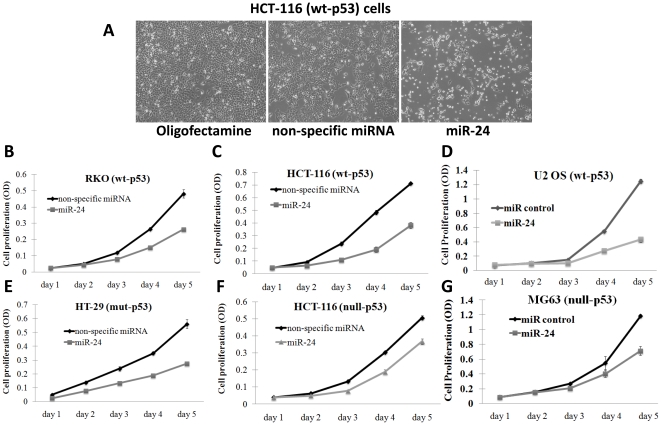
*MiR-24* inhibits anchorage-dependent cell proliferation independent of p53 status in six different cancer cell lines. (A) Morphological changes after the non-specific control-miR and *miR-24* over expression in HCT-116 (wt-p53) is demonstrated. (B–G) Six different cancer cell lines, with or without functional p53, were transfected with control miR (neg) or *miR-24* and cell number was determined. Upon *miR-24* transfection cell proliferation was inhibited approximately from 30% to 65% at day 5 as compared to the control (p<0.05, standard deviations are plotted as error bars on the graphs). Of interest, regardless of the p53 status of a cell, *miR-24* inhibited cell proliferation.

*MiR-24* is a highly conserved miRNA among species (Fig. S1). To assess the functional significance of *miR-24*, we evaluated its effect on cellular proliferation using six different cancer cell lines; four human colon cancer cell lines [HCT-116 (wt-p53), HCT-116 (null-p53), RKO (wt-p53) and HT-29 (mut-p53)] and two human osteosarcoma cell lines [U2OS (wt-p53) and MG63 (null-p53)]. A nonspecific *miR* was used as a negative control. Overexpression of *miR-24* suppressed cellular proliferation in all of the cell lines independent of p53 status (Fig. 1A–G) (p<0.05, standard deviations are plotted as error bars on the graph). The nonspecific control *miR* had no effect on cellular proliferation, suggesting that *miR-24* mediated inhibition of cellular proliferation is *miR-24*-specific.

**Figure 2:**
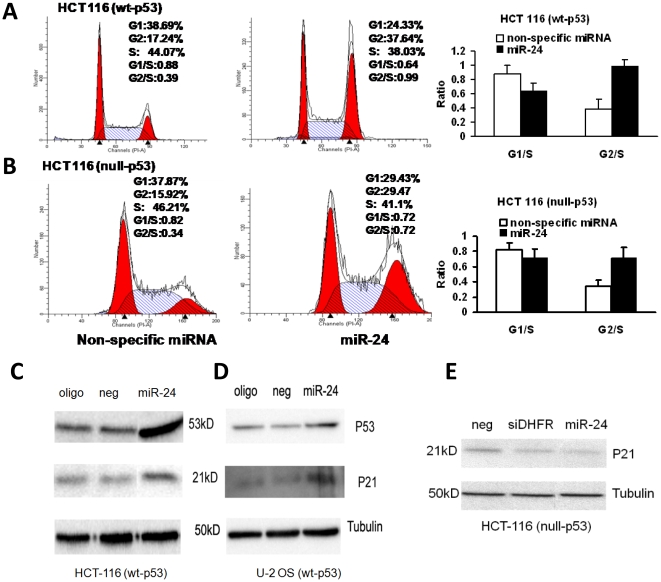
*MiR-24* induces a p53 independent G2-cell cycle arrest. (A, B) The bar graphs show the relative quantity of G1/S and G2/S ratio between the non-specific miRNA (negative control) and *miR-24* transfected cells. *MiR-24* transfection induced a G2-cell cycle arrest. (C, D) *miR-24* increases the expression of cell cycle control genes p53 and p21 in HCT 116 (wt-p53) and U-2 OS cells as determined by Western blotting. (E) In p53 null HCT-116 cells, independent of p21, *miR-24* inhibited the G2 arrest (B and E).

We next determined if the effect of *miR-24* on cellular proliferation was related to cell cycle control. The effect of *miR-24* on the cell cycle was analyzed by flow cytometry using HCT-116 (wt-p53) and HCT-116 (null-p53) cells transfected with a nonspecific control *miR* or *miR-24*. *MiR-24* increased the G_2_-S ratio in both colorectal cancer cell lines (Fig. 2A, B). This finding, together with the cell proliferation results (Fig. 1), confirmed that *miR-24* mediated inhibition of the cell-cycle is independent of p53 function.

### *MiR-24* overexpression affects cell cycle control genes

Induction of the p53-dependent cell cycle checkpoint control gene p21 triggers cell cycle arrest at both G_1_ and G_2_ phases [24]. To further analyze the cell cycle control genes involved in *miR-24* overexpression, we analyzed p53 and p21 expression by immunoblotting in HCT-116 (wt-p53), U-2OS (wt-p53) and HCT-116 cells (null-p53) (Fig. 2C–E). Ectopic expression of *miR-24* significantly increased the expression of p53 protein and p21 protein levels in HCT-116 (wt-p53) cells (Fig. 2C). *MiR-24* overexpression also induced p53 and p21 expression in the U2-OS osteosarcoma cell line (Fig. 2D). In contrast, a nonspecific-miR (neg) and oligofectamin (oligo) did not cause an increase in expression of p53 or p21 (Fig. 2C, D), suggesting that *miR-24*-mediated induction of p53 and p21 is specific to *miR-24* expression. However, in the HCT-116 cell line that lacked the p53 gene, p21 was not induced (Fig. 2E), indicating that in the absence of p53 function, miR-*24* can still induce cell cycle arrest without the involvement of p21.

### *MiR-24* regulates dihydrofolate reductase, a gene associated with cell proliferation, independent of p53 function

**Figure 3:**
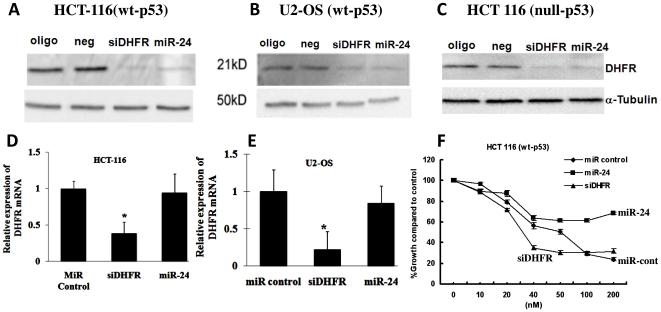
*MiR-24* regulates cell proliferation by regulating DHFR levels, independent of p53 status. *(A–C) miR-24* over-expression down-regulated DHFR protein levels in three cancer cell lines independent of p53 function; in colon cancer HCT 116 (wt-p53) (A), osteosarcoma cell line U2-OS (wt-p53) (B), and HCT-116 (null-p53) cancer cell lines (C). Oligofectamine alone (oligo) and non-specific miRNA (neg) were used as the negative controls. siRNA specific against DHFR (siDHFR) was the positive control. (D–E) The levels of DHFR mRNA in HCT-116 and U-2 OS cells were determined by real time qRT-PCR analysis, GAPDH was used as an internal standard for normalization (data are shown as mean±SD. *, *P*<0.05). (F) *miR-24* not only inhibited cell growth but also induced MTX resistance in a HCT-116 colorectal cancer cell line.

DHFR is an S-phase specific enzyme and its levels in the cell are associated with cellular proliferation [25]. Inhibition of DHFR activity by methotrexate affects tumor cell proliferation both in vitro and in cancer patients. *MiR-24* has a target site on DHFR 3′-UTR [22]. We reasoned that *miR-24*-mediated down regulation of DHFR may explain the anti-proliferative effect conferred by *miR-24* expression. To confirm this hypothesis, a *miR-24* precursor was transfected into cells of varying p53 function: HCT-116 (wt-p53), U2-OS (wt-p53), and HCT-116 (null-p53). A nonspecific *miR* was used as a negative control. DHFR *siRNA* was used as a positive control. The expression of DHFR protein was analyzed using Western blotting (Fig 3A–C). Overexpression of *miR-24* clearly decreased the expression of DHFR protein, with a potency that was comparable to control DHFR-siRNA, independent of the presence of functional p53. We also analyzed the expression level of DHFR mRNA using real-time qRT-PCR analysis in two cell lines. There was no reduction in DHFR mRNA expression associated with *miR-24* overexpression, whereas the decreased expression of DHFR by *siRNA* was clearly caused by mRNA degradation (Fig 3D, E). Thus, the *miR-24* suppression of DHFR expression was most likely regulated at the translational level independent of p53 status.

### *MiR-24* overexpression induces methotrexate resistance

**Figure 4:**
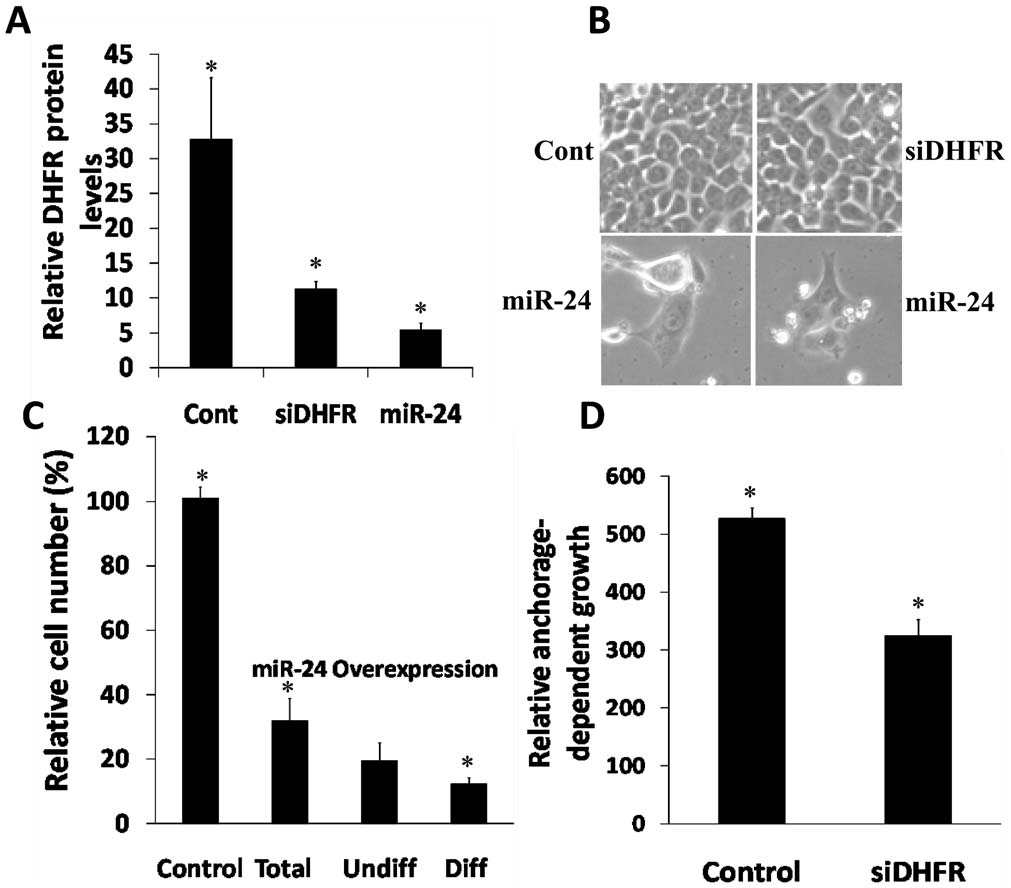
Overexpression of *miR-24* down regulated DHFR expression, reduced anchorage-dependent growth, and induced morphological changes resembling differentiation. (A) DHFR protein levels were down regulated upon over-expression of DHFR specific siRNA (siDHFR) (by three fold) and *miR-24* (by six fold). (B) *miR-24* over expression conferred morphological changes that resembled differentiated in a colorectal cancer cell line (HCT-116-wt-p53). The differentiated-like morphological changes were not observed in HCT-116 cells transfected with siDHFR and oligofectamine alone (Cont). (C) Due to a lack of well established differentiation markers, the morphological changes in *miR-24* transfected cells were quantitated by light microscopy. Although *miR-24* transfection resulted in approximately 70% reduction in cell proliferation, approximately 12% of the total transfected cells, and 38% of total surviving cells, showed a differentiation-like phenotype. (D) Of interest, siRNA specific to DHFR that down regulated DHFR levels in the cells by threefold also reduced anchorage-dependent growth of the HCT-116 colorectal cancer cell line, suggesting that DHFR levels in the cell are associated with cell proliferation (data are shown as mean±SD. *, *P*<0.05).

Generally, MTX treatment inhibits proliferation of rapidly dividing cancer cells without having a limited effect on the proliferation of differentiated cells. As *MiR-24* transfection has been shown to induce differentiation in cells, we anticipated that *miR-24* overexpression would confer resistance to antiproliferative drugs such as MTX. To test this hypothesis the effect of *miR-24* overexpression on MTX resistance was tested. We used HCT-116 (wt-p53) cells transfected with a *miR-24* mimic or non-specific miRNA, or siRNA against DHFR. MTX cytotoxicity was performed using previously published methods [22]. We observed that transfection of *miR-24* induced MTX resistance in HCT-116 cells, whereas cells transfected with siRNA specific to DHFR or control miRNA were sensitive to MTX as compared to *miR-24* transfected cells (Fig. 3F). Since miR-24 overexpression is associated with differentiation (see introduction for detail) (Fig. 1 and 4), we suggest that the slow growth and differentiation caused by *miR-24* overexpression contributes to MTX resistance.

### Overexpression of *miR-24* down regulated DHFR expression, reduced anchorage-dependent growth, and induced differentiation-like morphological changes in a colorectal cancer cell line

We quantitated DHFR protein levels, morphological changes, and anchorage-dependent growth upon transfection of a siRNA specific to DHFR and *miR-24*. *MiR-24* overexpression down regulated DHFR levels by approximately six fold as compared to Oligofectamine alone transfected cells (Fig. 4A), and conferred a morphological change resembling a differentiation-like phenotype in a colorectal cancer cell line (HCT-116-wt-p53). The differentiation-like morphological change was not observed in HCT-116 cells transfected with siDHFR and Oligofectamine alone (Cont) (Fig. 4B). Since differentiation markers for colorectal cancer cells are not well established, following *miR-24* transfection we used light-microscopy to quantitate the morphological changes in HCT-116 cells [34]. Although *miR-24* transfection reduced HCT-116 cell-proliferation by 70% (mostly cytotoxic effect), approximately 12% of the total transfected cells and 38% of the total surviving cells showed morphological changes that resembled differentiation (Fig. 4C). These morphological changes were observed in all six cancer cell lines tested, indicating these were independent of p53 function (data not shown). There are many reports that demonstrate that *miR-24* over expression is associated with a differentiated phenotype (see introduction for detail). However, further studies are required to confirm this observation in colorectal cancer cells by well characterizing and establishing differentiation markers, which is an active area of research in the laboratory. Of interest, we also observed that over expression of a DHFR specific siRNA (siDHFR) down-regulated DHFR levels by threefold as compared to Oligofectamine alone transfected cells (Fig. 4A), and also reduced anchorage-dependent growth of the HCT-116 colorectal cancer cell line by approximately two fold (Fig. 4D); these data suggest that changes in DHFR levels are directly associated with cell proliferation.

### A *miR-24* target site SNP in dihydrofolate reductase 3′UTR confers an ability on immortalized cells to form foci and growth in anchorage-independent fashion

**Figure 5:**
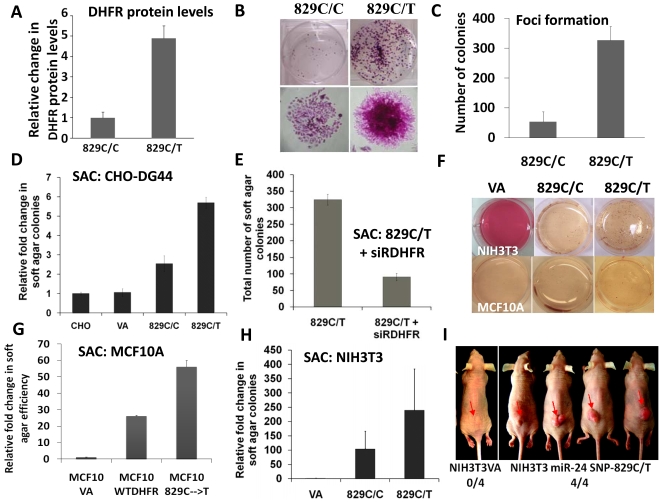
A loss-of-function *miR-24* target site SNP contributes to cellular transformation. (A) The *miR-24-TS-SNP* (829C→T) expressing CHO-DG44 cells over-expressed DHFR [22]. (B–C) DHFR over expression in CHO-DG44 cells due a loss of *miR-24* function results in an increase in colony forming ability and (D) show anchorage-independent growth in semi solid agar as compared to vector alone expressing cells (p-value>0.05) (Soft agar colony-forming assay is abbreviated as SAC). (E) Transfection of a siRNA specific to DHFR reduced the ability of *miR-24-TS-SNP* expressing cells to form soft agar colonies by threefold. (F-H) *miR-24-TS-SNP* expression in a human cell line - MCF10A (G) and two rodent cell lines - NIH3T3 (H) and RK3 (see Table S1) resulted in more soft agar colony formation as compared to the cells expressing the vector alone and DHFR with wt 3′UTR. (I) DHFR overexpression due to the *miR-24-TS-SNP* renders NIH3T3 cells tumorigenic when transplanted in nude mice.

DHFR is an S-phase enzyme and its levels are associated with cell proliferation. We demonstrated earlier that a *miR-24* target site SNP 829C→T (*miR-24-TS-SNP*) in the DHFR 3′ UTR results in DHFR overexpression due to loss of *miR-24* function (22). We next tested whether cells expressing a mutant *miR-24*-target site allele (C→T) that over expresses DHFR provides cells with a growth advantage. CHO DG44 cells were transfected with either DHFR cDNA containing the wt 3′UTR, or DHFR cDNA with the *miR-24-TS-SNP* in the DHFR 3′UTR. The *miR-24-TS-SNP* expressing cells had on average a nineteen-fold increase in DHFR mRNA, and four to five fold increase in levels of DHFR protein as compared to wild type DHFR expressing cells [22]. We found that cells overexpressing DHFR (>fourfold) (Fig. 5A) due to the *miR-24-TS-SNP* in the DHFR gene formed more colonies as compared to wt and vector alone cells (Fig. 5B, C). Of interest, the cells that overexpressed DHFR were morphologically distinct from the wt and the vector alone control cells (Fig. 5B). The colonies formed by the cells with the SNP resembled foci formation in monolayer cultures, characteristic of transformed cells. The wt and vector alone cells that expressed less DHFR grew in a monolayer and morphologically resembled normal DG44 cells under the microscope (Fig. 5B). Contact inhibition was lost in SNP expressing cells, resulting in foci formation with an increase in saturation density as compared to the vector control culture (Fig. 5B, C).

As a second test for the transformed phenotype we used a colony formation assay in soft agar. The ability of wt, *miR-24-TS-SNP* and vector alone expressing cells to form colonies was tested. We found that the cells with the *miR-24-TS-SNP* formed more colonies in soft agar and acquired an anchorage-independent phenotype with a six to seven fold increased efficiency as compared with the vector alone cells and three fold more than the cells that overexpressed wt DHFR with a wt 3′ UTR (Fig. 5D). Furthermore, to confirm that transformation was due to high levels of DHFR, we next explored the effect of DHFR knock down in the *miR-24-TS-SNP* expressing cells using a siRNA specific to DHFR. Transfection of the DHFR-specific siRNA in *miR-24-TS-SNP* expressing cells reduced the soft agar colony forming ability of the cells to threefold (Fig. 5E), suggesting that anchorage independent growth was specific to increased DHFR expression.

We next tested if overexpressed DHFR due to the *miR-24-TS-SNP* can also induce anchorage independent growth in two additional rodent cell lines (NIH3T3 cells and RK3-rat kidney cells) and human breast epithelial MCF10A cells, in addition to CHO cells. All three cell line are immortalized but not transformed. The wt DHFR, *miR-24-TS-SNP* and vector alone constructs were stably transfected into the three cell lines. All three cell lines overexpressing DHFR (>5-fold) formed more colonies in soft agar than the wt-expressing clones. No soft agar colonies were observed in the cells transfected with the vector alone (Fig. 5F–H, Table S1). Therefore, cells transfected with DHFR mRNA containing the *miR-24-TS-SNP* induced anchorage independent growth in three immortalized rodent cell lines (CHO, NIH3T3 and RK3) and an immortalized human cell line (MCF-10A).

### DHFR overexpression due to the SNP renders NIH3T3 cells tumorigenic in vivo

We next inoculated the *miR-24-TS-SNP* expressing NIH3T3 cells and vector alone expressing NIH3T3 cells subcutaneously on to the back of nude mice and tumor formation was monitored. All five animals that were inoculated with the SNP-expressing cells with high DHFR levels (>5-fold) formed tumors, whereas vector alone expressing cells did not form tumors (Fig. 5I). Hence DHFR overexpression due to a *miR-24-TS-SNP* makes NIH3T3 cells tumorigenic in nude mice.

### *MiR-24* is deregulated in human colorectal cancer tumors and there is a subset of tumors with reduced levels of *miR-24*

**Figure 6:**
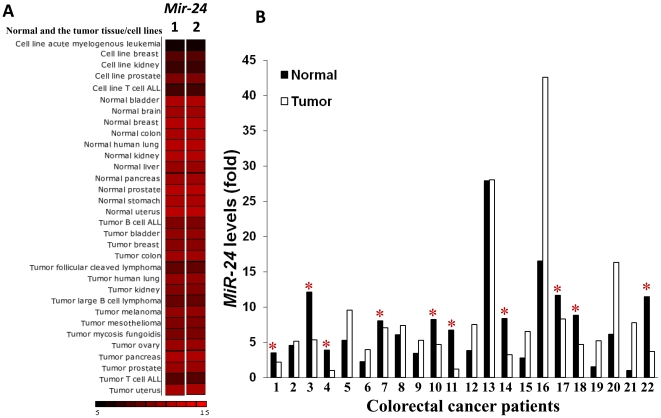
*MiR-24* is deregulated in human colorectal cancer tumors. (A) We investigated the *miR-24* expression, using miRNAMap-2 [20], in normal vs. cancer tissue and found that *miR-24* is expression was clearly detectable in normal cell lines/tissues but is down regulated in cancer cell line/tumor samples, as compared to the control cells/tissues (column 1, *miR-24-1*; column 2, *miR-24-2*). (B) *Mir-24* expression was assayed by Q-RTPCR in human colorectal specimens (24 paired colorectal normal mucosa and tumor samples) collected from patients undergoing surgical resection of primary colorectal adenocarcinoma.

We investigated miR-expression in normal vs. cancer tissue using miRNAMap-2 [26], and found that *miR-24* levels were frequently upregulated in normal but down regulated in cancer cell line/tumor samples (Fig. 6A). Further we tested *mir-24* expression in colorectal cancer tumors obtained from patients. Total RNA was isolated from a total of 48 colorectal clinical specimens (24 paired colorectal normal mucosa and tumor samples), collected from patients undergoing surgical resection of primary colorectal adenocarcinoma. *MiR-24* levels were determined using q-RT-PCR (see methods for details). Of clinical significance, we found that *miR-24* was down regulated in 45% of colorectal cancers as compared to the adjacent normal tissue.

## Discussion

Since their discovery in the early 2000s, miRNAs and their ability to regulate gene expression have revolutionized every corner of biological inquiry. Understanding how miRNAs orchestrate oncogenic progression may provide a better understanding of the disease and suggest novel therapeutic approaches [6]. In this report we demonstrate that *miR-24*, an abundant miRNA that is expressed at high levels in differentiated cells, has an anti-proliferative effect and mediates inhibition of the cell cycle independent of p53 and p21 function. *MiR-24* targets DHFR, a gene associated with cell proliferation, independent of p53 function. We demonstrate that a loss of function-*miR-24* target site polymorphism in the DHFR 3′UTR induces anchorage-independent growth in vitro and renders immortalized cells tumorigenic in nude mice. Of clinical significance, *miR-24* is deregulated in colorectal cancers from patients; 45% of the patients tested had down regulated *miR-24* expression in colorectal cancers as compared to the adjacent normal tissue. These data support the notion that *miR-24* functions as a p53-independent anti-proliferative miRNA and reexpression of *miR-24* may constitute a novel approach to arrest tumor development, at least in part by modulating DHFR expression.

We previously demonstrated that a loss of *miR-24* function polymorphism results in methotrexate resistance as a consequence of increased levels of DHFR mRNA and protein due to loss of translational regulation [22]. Of interest, we observed that over expression of *miR-24* rendered cells more resistant to MTX due to reduced cellular proliferation. Over expression of *miR-24* may affect expression of other proteins while the *miR-24-TS-SNP* phenotype has a more DHFR-specific effect. The loss of *miR-24* function target site polymorphism that results in DHFR over expression, increased cell proliferation and transformed immortalized cells suggests a novel role for *miR-24* as a tumor suppressor miRNA. Alternatively it can be anticipated that a *miR-24-TS-SNP* that can create miRNA-mediated repression of tumor-suppressor genes may also have an ability to transform the cell [27].

DHFR is preferentially synthesized in proliferating cells and is an essential enzyme for DNA synthesis and repair. High DHFR levels were found in human brain tumors, in hypoplastic myelodysplasia and in cisplatin-resistant human colon carcinoma cells [28]–[30]. Human-like DHFR was found to be encoded in Kaposi's sarcoma-associated herpes virus (KSHV), which is known to cause Kaposi's sarcoma and other hematological malignancies [31]. DHFR gene amplification was also correlated with the metastatic potential of rat adenocarcinoma cells [32]. Similarly, our work shows that over expression of DHFR leads to increased cellular proliferation and transformation of immortalized cells, and is regulated, at least in part, by *miR-24* miRNA. Transfection of DHFR cDNA and selection of cells with high DHFR levels also transformed immortalized cells in vitro and rendered them tumorigenic in vivo [25].

*MiR-24* regulates the G2/S phase of the cell cycle independent of p53 and p21 function, which in part can be explained by its ability to regulate DHFR translation. However, miRNAs have the ability to up-regulate or down-regulate several essential cellular enzymes and push the cell into cycle. It has been reported recently that *miR-24* can suppress the expression of E2F2, Myc and other cell cycle control genes and trigger cell cycle arrest [23]; however cell lines used were either mutant or p53 deleted [23]. In this study, we demonstrate that p21 levels in the cell are increased upon *miR-24* overexpression only in the presence of p53.

Expression of DHFR in S-phase is required for DNA biosynthesis; this is consistent with the finding that the expression level of *miR-24* was high in G1 and G2/M but low in S phase [23]. Deregulations of both *miR-24* sites were found to be associated with CLL [8]. We demonstrate that *miR-24* levels are deregulated in tumors obtained from colorectal cancer patients. Taken together these data indicate that *miR-24* is an important regulator of cell proliferation and reexpression of *miR-24* may have therapeutic anticancer value.

The *miR-24-TS-SNP* 829C→T occurs at a 14.2% allelic frequency in the Japanese population, and may predispose cells for cellular transformation following other events in a cell. MiRNA-polymorphisms (miR-polymorphisms or miRSNPs) are a novel class of functional polymorphisms present in the human genome [33], [34]. Cumulative evidences now suggest that genetic variations in miRNAs and are involved in the progression and prognosis of diseases, including neurological disorders and cardiovascular disorders and cancer [35], [36]. By affecting miRNA target function, miR-polymorphisms can potentially affect the expression of several downstream genes and related pathways in a cell [36]. We demonstrate that a loss of miR-24 function-SNP that results in DHFR over expression and MTX resistance, following other events in a cell, can also predispose immortalized cells for transformation. Further inquiry in to related ethnic groups as to its presence, and its effect on treatment outcome and or toxicity will be of importance.

In summary we propose a novel role for the miRNA *miR-24* as an anti-proliferative miRNA, independent of p53 function, by showing that it targets a pro-proliferation gene DHFR. Loss of *miR-24* function, due to a SNP in the 3′UTR of DHFR leads to overexpression of DHFR mRNA and protein and transformation of immortalized cells.

## Materials and Methods

### Ethics Statement

Patient consent was obtained in writing according to institutional regulations. The studies were approved by the Ethics Committee of the University of Ulm (Ulm, Germany) and by the University of Medicine and Dentistry of New Jersey (UMDNJ) Institutional Review Board (IRB) in writing [IRB # 0220080331, written consent was obtained on 1/22/09].

### Cell culture and growth media

The human osteosarcoma cell lines U-2 OS, MG63, RKO and HT-29 were obtained from the American Type Culture Collection (ATCC). The human colon cancer cell lines HCT 116 (wt-p53) and HCT 116 (null-p53) were a gift from Professor Bert Vogelstein (The Johns Hopkins University) and were maintained in McCoy's 5A medium (Gibco Laboratories) supplemented with 10% dialyzed fetal bovine serum (HyClone Laboratories). MTX was purchased from Sigma-Aldrich. NIH/3T3, Rat kidney cells (RK3) were purchased from ATCC and grown in Dulbelcco's modified Eagle's medium (DMEM) with 10% fetal bovine serum (FBS), 1% penicillin/streptomycin, 1% l-glutamine (Gibco-BRL).

### Transfections of *miR-24* and siRNAs

RKO, HT-29, U2-OS, MG63, HCT-116 (wt-p53) and HCT-116 (null-p53) cells (2×10^5^) were plated in six-well plates and transfected with 100 nM of either *miR-24* or non-specific miRNA (Ambion) after 24 h by Oligofectamine (Invitrogen) according to the manufacturer's protocols. siRNA against DHFR was purchased from Dharmacon and transfected with Oligofectamine (Invitrogen) at a final concentration of 100 nM.

### RNA Isolation

Total RNA, including miRNA, was isolated from the *miR-24* transfected cell lines (24 h after transfection) and from clinical colorectal cancer samples using TRIzol reagent, according to the manufacturer's instructions (Invitrogen).

### Real Time qRT-PCR Analysis

cDNA synthesis was carried out with the High Capacity cDNA synthesis kit (Applied Biosystems). For mRNA expression the PCR primers and probes for DHFR and internal control gene GAPDH were purchased from Applied Biosystems. Real-time quantitative reverse transcription-PCR (qRT-PCR) analysis was performed on an Applied Biosystems 7500 Real-Time PCR System. The miRNA sequence-specific RT-PCR primers for *miR-24* and endogenous control RNU6B were purchased from Ambion. The gene expression Δ*C*_T_ values of miRNAs from each clinical sample were calculated by normalizing with internal control RNU6B and relative quantitation values were plotted. Sample with the lowest Δ*C*_T_ value of *miR-24* was set as 1 to generate relative expression values using 2^−dd*C*T^ method [37].

### Cell Cycle Analysis

HCT 116 (wt-p53) and HCT 116 (null-p53) cells were transfected with *miR-24* mimic, non-specific miRNA or siRNA against DHFR described as above. At 36 h after transfection, cells were harvested and resuspended at 0.5−1×10^5^ cells/ml in modified Krishan buffer containing 0.1% sodium citrate and 0.3% NP-40 and kept at 4°C. Before being analyzed by flow cytometry, cells were treated with 0.02mg/ml RNase H and stained with 0.05mg/ml propidium iodide (Sigma).

### Western blot analysis and antibodies

At 48 h after transfection with *miR-24* mimic or non-specific miRNA, the cells were scraped and lysed in RIPA buffer (Sigma). The primary antibodies included mouse anti-DHFR mAb (1∶250, BD Bioscience), mouse anti-p53 mAb (1∶1000, DO-1), mouse anti-p21 mAb (1∶1000, F-5), and mouse anti-α-tubulin mAb (1∶1000, TU-02) from Santa Cruz Biotechnology. Oligofectamine alone and non-specific miRNA were used as the negative controls. siRNA against DHFR was used as the positive controls. Bands were quantitated using National Institute of Health's ImageJ software.

### MTX Chemosensitivity

HCT 116 (wt-p53) cells were plated in 96-well plates at 1×10^3^ cells/well in triplicate after transfected with *miR-24* mimic, non-specific miRNA, or siRNA against DHFR in 100 µl of medium. Twenty-four hours later, MTX in 100 µl medium ranged from 10–200 nM was added, and incubated for 72 h. WST-1 (Roche Applied Science) was added to each well (10 µl). After 2 h incubation, absorbance was measured at 450 and 630 nm respectively. Non-specific miRNA was used as the negative control, and siRNA specific for DHFR was positive controls.

### Cloning and site directed mutagenesis cell transfections and generation of stable clones

Wild type DHFR was cloned in a pCDNA3.1 vector and the 829C→T mutation was created using QuickChange site directed mutagenesis kit (Stratagene, La Jolla, CA) as described previously [22]. The vector alone wt and mutant constructs were transfected into the CHO-DG44, NIH3T3, RK3 and in MCF10A cells. The cells were transfected, selected with G418, and were picked and expanded as cell lines [22].

### Growth, morphological changes and anchorage-independent growth assays

For cell proliferation analysis on the six cancer cell lines was performed by plating the cells in 96-well plates in triplicate at 1×10^3^ cells/well after transfection with *miR-24* mimic, non-specific miRNA, or siRNA against DHFR (*n* = 3). Cells were cultured for 24, 48, 72, 96 h. The absorbance at 450 and 630 nm was measured after incubation with 10 µl of WST-1 for 2 h. Differentiation-like morphological changes in HCT-116 cells upon miR-24 transfection was quantitated by light-microscopy [38]. Anchorage-independent growth was carried out by plating 5×10^4^ cells per well in 6-well plates in triplicates [39]. After 4–6 weeks incubation the cells were fixed with methanol and acidic acid solution (10%), and stained with ethanol (20%), crystal violet (0.4%); the colonies were scored and large and small colonies were counted. The growth of the clones was assayed by platting 50,000 cells per well in 12-well plates in triplicates and counted at 1–6 days.

### Xenograft studies in nude mice

Tumorigenicity of DHFR over expressing NIH3T3 and RK3 cells was assayed by tumor formation in nude mice. Cells expressing DHFR and vector alone constructs were expanded for two passages without selection. Ten million cells in a total volume of 100µl in growth medium were injected subcutaneously into the backs of 5 to 6-week-old NCR *nu/nu* mice (Taconic Farms Inc, USA). The growths of tumors were followed three times weekly after the inoculation.

### Clinical Samples

A total of 48 snap-frozen colorectal patient specimens were selected (24 paired colorectal normal mucosa and tumor samples). These patients had undergone surgical resection of primary colorectal adenocarcinoma at the Department of Visceral and Transplantation Surgery, University of Ulm, Germany. The colorectal cancer studies were approved by the Ethics Committee of the University of Ulm (Ulm, Germany) and by the University of Medicine and Dentistry of New Jersey (UMDNJ) Institutional Review Board (IRB) in writing [IRB # 0220080331, written consent was obtained on 1/22/09]. Patient consent was obtained in writing from every patient according to the institutional regulations. The characteristics of these patients are described as previously [40]. RNA was isolated using the same method described above (via supra).

### Statistical analysis

All experiments were repeated at least twice. Statistical significance was evaluated by Student's t test (two tailed) comparison between two groups of data. Asterisks indicate significant differences of experimental groups compared with the corresponding control condition. Statistical analysis was done using GraphPad Prism software (GraphPad, Inc.). Variance ratio test (F-test) was used to compare the variances of *miR-24* levels in normal vs cancer tissue. The statistically significant difference in expression level between tumor and normal tissues was calculated using a paired Wilcoxon test, and the statistical analysis was performed by MedCalc® 10.0.2 (MedCalc software). Differences were considered statistically significant at *p*<0.05.

## Supporting Information

Figure S1 MiR-24 precursor and it's conservation. A) miR-24 stem-loop precursor is shown (B) miR-24 are well conserved among species, from mouse to humans.
(1.24 MB TIF)

Table S1 Anchorage independent growth ability of miRSNP expressing cells in three different cell types.
(0.03 MB DOC)
