# Supplementary figures and images for: MiR-24 Tumor Suppressor Activity Is Regulated Independent of p53 and through a Target Site Polymorphism
Source: PLoS One. 2009 Dec 24;4(12):e8445. doi: 10.1371/journal.pone.0008445 (PMC2794546; doi:10.1371/journal.pone.0008445)

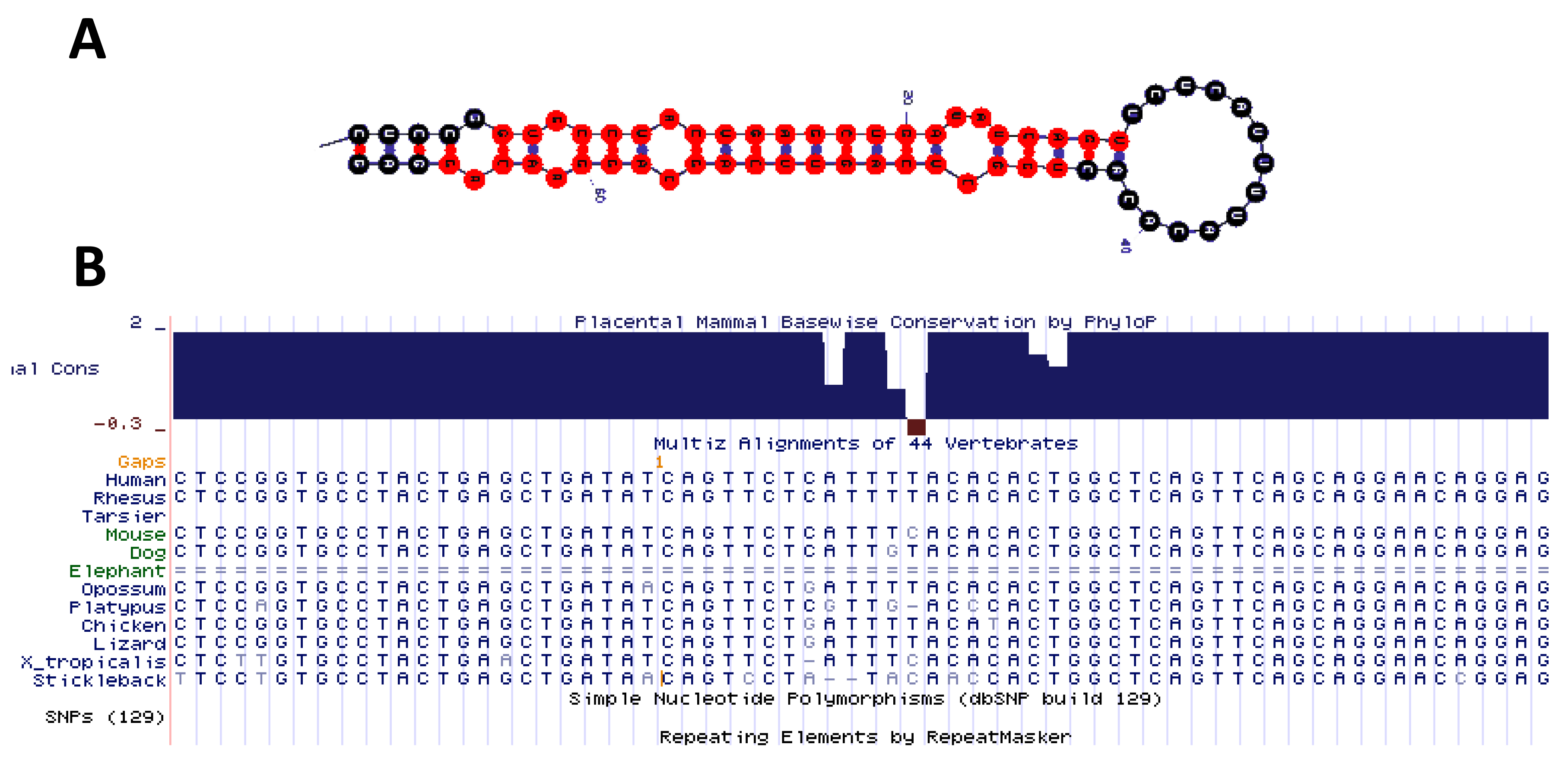

Supplement: Figure S1 — MiR-24 precursor and it's conservation. A) miR-24 stem-loop precursor is shown (B) miR-24 are well conserved among species, from mouse to humans. (1.24 MB TIF) [file pone.0008445.s001.tif]
