# Supplementary material for: MiR-24 Tumor Suppressor Activity Is Regulated Independent of p53 and through a Target Site Polymorphism
Source: PLoS One. 2009 Dec 24;4(12):e8445. doi: 10.1371/journal.pone.0008445 (PMC2794546; doi:10.1371/journal.pone.0008445)
Supplement: Table S1 — Anchorage independent growth ability of miRSNP expressing cells in three different cell types. (0.03 MB DOC) [file pone.0008445.s002.doc]

**Table S1. Anchorage independent growth ability of miRSNP expressing cells in three different cell types.**

| **Cell line** | **Ave. Small** | **Ave. Large** | **Average total** | **% efficiency in agar** |
| --- | --- | --- | --- | --- |
|  | **Colonies** | **Colonies** | **Colonies** |  |
| NIH3T3 DHFR 829C/C | 170 | 14 | 184 | 0.184 |
| NIH3T3 DHFR 829C/T | 330 | 40 | 369 | 0.369 |
| NIH3T3 VA | 1 | 0 | 1 | 0.001 |
| RK3 DHFR 829C/C | 9 | 0 | 10 | 0.010 |
| RK3 DHFR 829C/T | 39 | 13 | 52 | 0.052 |
| RK3 VA | 0 | 0 | 0 | 0.000 |
| MCF10A DHFR 829C/C | 357 | 26 | 383 | 0.184 |
| MCF10A DHFR 829C/T | 821 | 99 | 821 | 0.369 |
| MCF10A VA | 15 | 0 | 15 | 0.001 |
|  |  |  |  |  |

**Foot note:** Anchorage independent growth of the cells was assayed by colony formation in semisolid medium as described previously (39) (see methods). VA- cells expressing vector alone; DHFR 829C/C- cells expressing the wt DHFR 3’UTR; DHFR C/T- cells expressing the mutant DHFR UTR.
